# Fecal Contamination of Drinking-Water in Low- and Middle-Income Countries: A Systematic Review and Meta-Analysis

**DOI:** 10.1371/journal.pmed.1001644

**Published:** 2014-05-06

**Authors:** Robert Bain, Ryan Cronk, Jim Wright, Hong Yang, Tom Slaymaker, Jamie Bartram

**Affiliations:** 1 The Water Institute, University of North Carolina at Chapel Hill, Chapel Hill, North Carolina, United States of America; 2 University of Southampton, Southampton, United Kingdom; 3 WaterAid UK, London, United Kingdom; University of East Anglia, United Kingdom

## Abstract

**Background:**

Access to safe drinking-water is a fundamental requirement for good health and is also a human right. Global access to safe drinking-water is monitored by WHO and UNICEF using as an indicator “use of an improved source,” which does not account for water quality measurements. Our objectives were to determine whether water from “improved” sources is less likely to contain fecal contamination than “unimproved” sources and to assess the extent to which contamination varies by source type and setting.

**Methods and Findings:**

Studies in Chinese, English, French, Portuguese, and Spanish were identified from online databases, including PubMed and Web of Science, and grey literature. Studies in low- and middle-income countries published between 1990 and August 2013 that assessed drinking-water for the presence of *Escherichia coli* or thermotolerant coliforms (TTC) were included provided they associated results with a particular source type. In total 319 studies were included, reporting on 96,737 water samples. The odds of contamination within a given study were considerably lower for “improved” sources than “unimproved” sources (odds ratio [OR] = 0.15 [0.10–0.21], I^2^ = 80.3% [72.9–85.6]). However over a quarter of samples from improved sources contained fecal contamination in 38% of 191 studies. Water sources in low-income countries (OR = 2.37 [1.52–3.71]; *p*<0.001) and rural areas (OR = 2.37 [1.47–3.81] *p*<0.001) were more likely to be contaminated. Studies rarely reported stored water quality or sanitary risks and few achieved robust random selection. Safety may be overestimated due to infrequent water sampling and deterioration in quality prior to consumption.

**Conclusion:**

Access to an “improved source” provides a measure of sanitary protection but does not ensure water is free of fecal contamination nor is it consistent between source types or settings. International estimates therefore greatly overstate use of safe drinking-water and do not fully reflect disparities in access. An enhanced monitoring strategy would combine indicators of sanitary protection with measures of water quality.

*Please see later in the article for the Editors' Summary*

## Abstract 2

Robert Bain and colleagues conduct a systematic review and meta-analysis to assess whether water from “improved” sources is less likely to contain fecal contamination than “unimproved” sources and find that access to an “improved source” provides a measure of sanitary protection but does not ensure water is free of fecal contamination.

*Please see later in the article for the Editors' Summary*

## Editors' Summary

**Background:**

Access to clean water is fundamental to human health. The importance of water to human health and wellbeing is encapsulated in the Human Right to Water, reaffirmed by the United Nations in 2010, which entitles everyone to “sufficient, safe, acceptable and physically accessible and affordable water for personal and domestic uses.” A step towards such universal access to water is Millennium Development Goal (MDG) target 7c that aims to halve the proportion of the population without sustainable access to safe drinking-water. One of the indicators to help monitor progress towards this target used by the Joint Monitoring Project (JMP—an initiative of the World Health Organization and UNICEF) is “use of an improved source.” Improved sources include piped water into a dwelling, yard, or plot, or a standpipe, borehole, and protected dug well. Unimproved sources are those that do not protect water from outside contamination, such as unprotected wells, unprotected springs, and surface waters.

**Why Was This Study Done?:**

While this simple categorization may reflect established principles of sanitary protection, this indicator has been criticized for not adequately reflecting safety, suggesting that reported access to safe water might be overestimated by billions of people by not accounting for microbial water safety or more fully accounting for sanitary status. So the researchers conducted a systematic review and meta-analysis to investigate whether water from improved sources is less likely to exceed health-based guidelines for microbial water quality than water from unimproved sources and to what extent microbial contamination varies between source types, between countries, and between rural and urban areas.

**What Did the Researchers Do and Find?:**

The researchers comprehensively searched the literature to find appropriate studies that investigated fecal contamination of all types of drinking-water in low and middle-income countries. The researchers included studies that contained extractable data on *Escherichia coli* or thermotolerant coliform (the WHO recommended indicators of fecal contamination) collected by appropriate techniques. The authors also assessed studies for bias and quality and used a statistical method (random effects meta-regression) to investigate risk factors and settings where fecal contamination of water sources was most common.

Using these methods, the authors included 319 studies reporting on 96,737 water samples. Most studies were from sub-Saharan Africa, southern Asia, or Latin America and the Caribbean. They found that overall, the odds (chance) of contamination within a given study were considerably lower for “improved” sources than “unimproved” sources (odds ratio = 0.15). However, in 38% of 191 studies, over a quarter of samples from improved sources contained fecal contamination. In particular, protected dug wells were rarely free of fecal contamination. The researchers also found that water sources in low-income countries, and rural areas were more likely to be contaminated (both had odds ratios of 2.37).

**What Do These Findings Mean?:**

These findings show that while water from improved sources is less likely to contain fecal contamination than unimproved sources, they are not consistently safe. This study also provides evidence that by equating “improved” with “safe,” the number of people with access to a safe water source has been greatly overstated, and suggests that a large number and proportion of the world's population use unsafe water. As studies rarely reported stored water quality or sanitary risks, the accuracy of these findings may be limited. Nevertheless, the findings from this study suggest that the Global Burden of Disease 2010 may greatly underestimate diarrheal disease burden by assuming zero risk from improved water sources and that new indicators are needed to assess access to safe drinking water. Therefore, greater use should be made of other measures, such as sanitary inspections, to provide a complementary means of assessing safety and to help identify corrective actions to prevent water contamination.

**Additional Information:**

Please access these Web sites via the online version of this summary at http://dx.doi.org/10.1371/journal.pmed.1001644.

- *PLOS Medicine* has a published series on water and sanitation
- The WHO/UNICEF Joint Monitoring Programme for Water Supply and Sanitation has extensive information including data and country files
- UN Water  is the inter-agency coordination mechanism for all freshwater and sanitation related matters
- The UN also provides information on the human right to water and sanitation

## Introduction

The importance of water to human health and wellbeing is encapsulated in the Human Right to Water and Sanitation, which entitles everyone to “sufficient, safe, acceptable physically accessible and affordable water for personal and domestic uses” [1], as reaffirmed by the United Nations General Assembly and Human Rights Council in 2010 [2]. Millennium Development Goals (MDGs) Target 7c aims “to halve the proportion of the population without sustainable access to safe drinking-water …” [3], a step towards universal access. “Use of an improved source” was adopted as an indicator for monitoring access to safe drinking-water globally (Table 1) and relies on national censuses and nationally representative household surveys as the primary sources of data.

**Table 1:** Types of improved source and the estimated proportion of the global population using these as their primary source of drinking-water.

Source Category^a^	Description	Global Population Using Water Source in 2010^b^ (%)		
		Urban	Rural	Total
Household or yard connection	Piped water into dwelling, also called a household connection, is defined as a water service pipe connected with in-house plumbing to one or more taps. Piped water to yard/plot, also called a yard connection, is defined as a piped water connection to a tap placed in the yard or plot outside the dwelling.	80	29	54
Standpipe	Public tap or standpipe is a public water point from which people can collect water. A standpipe is also known as a public fountain or public tap. Public standpipes can have one or more taps and are typically made of brickwork, masonry, or concrete.	6	8	7
Borehole	Tubewell or borehole is a deep hole that has been driven, bored, or drilled, with the purpose of reaching groundwater supplies. Boreholes/tubewells are constructed with casing, or pipes, which prevent the small diameter hole from caving in and protects the water source from infiltration by runoff water.	8	30	18
Protected dug well	Protected dug well is a dug well that is protected from runoff water by a well lining or casing that is raised above ground level and a platform that diverts spilled water away from the well. A protected dug well is also covered, so that bird droppings and animals cannot fall into the well.	2^c^	10^c^	6^c^
Protected spring	The spring is typically protected from runoff, bird droppings, and animals by a “spring box,” which is constructed of brick, masonry, or concrete and is built around the spring so that water flows directly out of the box into a pipe or cistern, without being exposed to outside pollution.	<1^c^	3^c^	2^c^
Rainwater	Rainwater refers to rain that is collected or harvested from surfaces (by roof or ground catchment) and stored in a container, tank, or cistern until used.	<1	2	1

Source: UNICEF/WHO [5].

a Households using bottled water as their primary source of water for drinking are generally considered to use an improved source if one of the above sources is used for washing and cooking. The JMP estimates that 6% of the urban population and 1% of the rural population primarily use bottled water as their source of drinking-water.

b An estimated 3% of the global population use surface waters and a further 8% use “other unimproved sources” such as tanker trucks, unprotected dug wells, and unprotected springs.

c Published estimates include do not distinguish protected and unprotected. In the absence of data we assumed half are protected.

The Joint Monitoring Programme for Water Supply and Sanitation (JMP) of WHO/UNICEF categorizes a drinking-water source type as improved if “by nature of its construction or through active intervention, [it] is protected from outside contamination, in particular from contamination with faecal matter” [4]. Improved source types include piped water into dwelling, yard, or plot, standpipe, borehole, protected dug well or spring, and rainwater. Unimproved source types are those that do not protect water from outside contamination (unprotected wells, unprotected springs, surface waters, and tanker trucks). While the categorization reflects well-established principles of sanitary protection, on announcing that the target had been met in 2010, the JMP cautioned that the MDG indicator does not take water quality measurements into account [5]. The indicator has been criticized for not adequately reflecting safety [6]–[8], with some estimates suggesting that reported access to safe water might be overestimated by billions of people [9],[10], by not accounting for microbial water safety [8] or more fully accounting for sanitary status [9].

Diseases related to contamination of drinking-water constitute a major burden on public health. The principal risk to health is from ingestion of water contaminated with feces containing pathogens that cause infectious diseases such as cholera and other diarrheal diseases, dysenteries, and enteric fevers [11],[12]. The burden of water-related disease varies according to context and is highest in low-income settings where diarrhea remains a leading cause of child deaths [13]. Systematic reviews of epidemiological evidence from intervention studies [14]–[18], and especially outbreak investigations [19],[20], suggest drinking-water quality plays an important role in fecal-oral transmission, though the magnitude of the effect has been contested owing to a limited number of blinded trials [21]. It is difficult to isolate the effects of one component of the multiple and interrelated fecal-oral pathways, which are highly context-specific.

WHO publishes widely recognized Guidelines for Drinking-water Quality (GDWQ) (4th edition) that include criteria for assessing health risks and setting targets for improving water safety [12]. Direct measurement of pathogens is complex but techniques for assessing fecal contamination using fecal indicator bacteria (FIB) are well established and widely applied. The WHO GDWQ recommend using *E. coli*, or alternatively thermotolerant coliform (TTC), and new enzymatic methods have made quantification simpler, cheaper, and more robust [22],[23]. The WHO GDWQ recommend that *E. coli*, or alternatively TTC, be used in assessing fecal contamination of drinking-water [12]. The WHO guideline value for *E. coli* (“none detected in any 100-ml sample”) [12] is reflected in the standards of most OECD member states and low- and middle-income countries (LMICs). The WHO GDWQ further suggest the use of a risk classification to prioritize interventions as higher levels of indicator organisms are generally indicative of greater levels of fecal contamination. A commonly used risk classification is based on the number of indicator organisms in a 100 ml sample, which includes: <1, “very low risk”; 1–10, “low risk”; 10–100, “medium risk”; >100, “high risk” or “very high risk” [24],[25]. However FIB are imperfect and their level does not necessarily equate to risk [26]; since quality varies both temporally and spatially, occasional sampling may not accurately reflect actual exposure.

A complementary approach in safety assessment is the identification of hazards and preventative risk management measures through “sanitary inspection” of a water source and its surroundings [24],[27]. The improved source indicator is in effect a very simplified form of sanitary inspection. Like FIB, sanitary inspections have long been a tool in assessing drinking-water safety. In 1904, Prescott and Winslow stated, “[t]he first attempt of the expert called in to pronounce upon the character of a potable water should be to make a thorough sanitary inspection…” [28]. Standardized forms can be used to assess sanitary risk and derive a summary measure, the sanitary risk score. These forms typically include questions about the integrity of protective elements, such as fencing or well covers, and the proximity of hazards such as latrines; forms are available for different types of water source. Like water quality, some sanitary risk factors may vary spatially and temporally. The approach can be combined with microbiological analysis, either to yield a risk cross-tabulation [24],[25] or as a part of a more detailed Water Safety Plan [29].

In January 2012, WHO and UNICEF established working groups to develop targets and indicators for enhanced global monitoring of drinking-water, sanitation, and hygiene post-2015. The water working group proposed to continue using the improved water source classification as part of a revised set of indicators for assessing progressive improvements in service [30]. This review was commissioned to assist the group in evaluating the evidence linking improved source types and health-related indicators of water quality. The following specific questions were considered in order to determine the potential and limits of classification by source type in assessing safety in future global reporting: (i) Is water from improved sources less likely to exceed health-based guidelines for microbial water quality than water from unimproved sources? (ii) To what extent does microbial contamination vary between source types, between countries, and between rural and urban areas? (iii) Are some types of water source associated with higher risk scores as assessed by sanitary inspection?

## Methods

We conducted a systematic review of studies of fecal contamination of drinking-water in LMICs in adherence with PRISMA guidelines (Text S1) [31]. The protocol for the review is described in Protocol S1.

### Search Strategy

Studies were identified from both peer-reviewed and grey literature. To identify peer-reviewed literature, the topic “water quality” was combined with terms to restrict the search to drinking-water and either a measure of microbial water quality (e.g., “coli”) or sanitary risk (e.g., “sanitary inspection”). We further restricted the search to LMICs using a list of country names based on the MDG regions [32]. Online databases were searched including PubMed, Web of Science, and the Global Health Library. Grey literature was sourced from a variety of sites including those used in previous drinking-water–related reviews [33]–[35]. Translated search terms (Chinese, French, Portuguese, and Spanish) were used to identify additional studies. An email requesting submissions of relevant studies was distributed to water sector professional networks. We searched bibliographies of included studies and contacted authors where full texts could not be obtained through other means. Searches were conducted between 7th January and 1st August 2013.

### Eligibility and Selection

**Figure 1:**
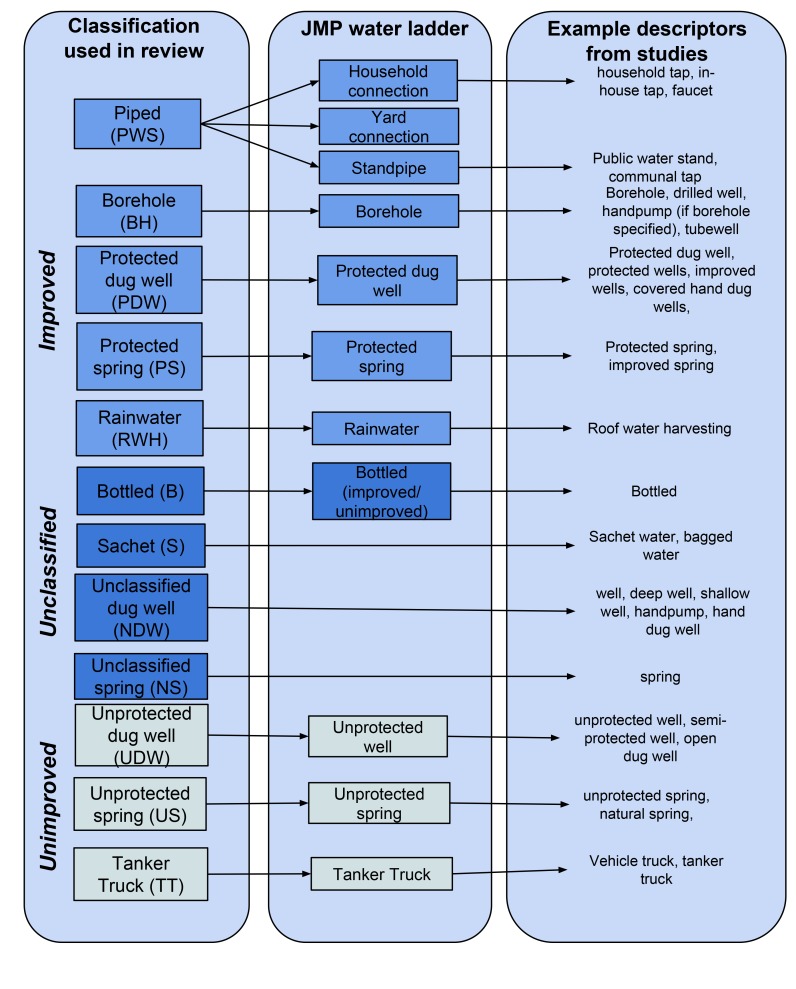
Matching drinking-water source types to the classification used by the Joint Monitoring Programme.

Studies were included in the review provided they: reported on water quality, at either the point of collection or consumption, from sources used for drinking that would not be classified as surface waters by the JMP; contained extractable data on TTC or *E. coli* with sample volumes not less than 10 ml; were published between January 1990 (the baseline year for MDG targets) and August 2013; included results from at least ten separate water samples from different water sources of a given type or, in the case of piped systems, individual taps, and in the case of packaged waters, brands; reported data from LMICs as defined by the MDG regions [32] (thereby excluding 55 high-income countries, comprising 18.1% of the global population in 2010 [36]); were published in languages spoken by at least one author (Chinese, English, French, Portuguese, or Spanish); and included sufficient detail about the water sources and associated results with a water source with sufficient detail to be categorized (refer to Figure 1 for details). Other indicators such as coliphage and direct pathogen detection are not as widely used and are not included in this review [37]. We did not include studies that only assessed surface waters as these are generally considered unfit for drinking. We included bottled water and sachet water that do not form part of the JMP improved source classification (which is concerned with the household's primary source of water for drinking, cooking, and personal hygiene [38]) but are nonetheless important sources of drinking-water in many countries.

Independent primary screening of English language titles and abstracts for studies was conducted by two authors (RB and RC). If any reviewer selected a study, we referred to the full text. Data from eligible studies were extracted into a standardized spreadsheet and 10% of the English language texts were subjected to independent quality control by a second author (RB and RC). Screening and extraction of data in other languages was conducted by one author (RB or HY).

### Data Extraction and Matching

Where possible we extracted or calculated the following information for each type of water source in the studies: (i) total number of samples and proportion containing *E. coli* or TTC; (ii) proportion of samples within microbial risk categories (<1 or not detected, 1–10, 10–100, and >100 *E. coli* or TTC per 100 ml); (iii) geometric mean, mean, or median levels of *E. coli* or TTC; and (iv) risk categories according to the sanitary inspection (“low,” “medium,” “high,” and “very high” risk) as reported in the studies. For intervention studies (other than the provision of an improved source, for example the protection of unprotected springs), estimates could be based on either the baseline or control group; when both were available we used whichever had the largest sample size. For studies reporting both *E. coli* and TTC, we used only the *E. coli* results. Where repeated measures were taken at the same source and data permitted we extracted the lowest compliance level (e.g., wet season data) with WHO Guideline values as well as the overall proportion of samples containing FIB. We identified countries as “low,” “lower middle,” “upper middle,” and “high” income using the 2013 World Bank classification [39]. We recorded whether studies took place during or shortly after emergencies or natural disasters and if they were in non-household settings such as schools and health facilities. We identified additional study characteristics expected to influence water quality, including the setting (urban/rural), season (wet/dry or period of sampling), and study design [34].

Each type of water source in a given study was classified as improved or unimproved and matched to a specific water source type following the classification used in household surveys including the Demographic and Health Surveys [38]. We recorded whether samples had been taken directly from the water source or after storage, for example in the home. Where the appropriate match could not be determined, our approach differed depending on the type of source. We grouped groundwater sources from studies that did not distinguish between protected and unprotected (unclassified dug well, unclassified spring) and we created groups for studies of other sources such as bottled and sachet water. Further information about the matching is available in Figure 1.

### Study Quality and Bias

Studies were rated for quality on the basis of the criteria summarized in Table 2. A quality score between 0 and 13 for each study was determined on the basis of the number of affirmative responses. We also categorized studies on the basis of anticipated susceptibility to bias in estimating the compliance to health-based guidelines and the extent of microbial contamination; our categories were: case-control or cohort, intervention, diagnostic study, cross-sectional survey, and longitudinal survey. Any study of at least 6 months duration and more than two samples at each water point was categorized as longitudinal. We identified studies where authors indicated whether selection was intended to be representative or selection had been randomized.

**Table 2:** Quality criteria used to assess studies of microbial water quality.

Criterion	Question
Selection described	Do the authors describe how the water samples were chosen, including how either the types of water source or their users were selected?
Selection representative	Did the authors detail an approach designed to provide representative picture water quality in a given area?
Selection randomized	Was sampling randomized over a given study area or population?
Region described	Does the study report the geographic region within the country where it was conducted?
Season reported	Were the seasons or months of sampling reported?
Quality control	Were quality control procedures specified or referred to?
Method described	Are well-defined and appropriate methods of microbial analysis described or referenced?
Point of sampling	Was the point at which water was sampled well defined? (For example whether the water was collected from within a household storage container or directly from a water source)
Handling described	Are sample handling procedures described, including sample collection, transport method, and duration?
Handling minimum criteria	Does sample handling and processing meet the following criteria: transport on ice or between 2–8°C, analysis within 6 hours of collection, and specified incubation temperature?
Accredited laboratory	Was the microbial analysis conducted in an accredited laboratory setting?
Trained technician	Do the authors state whether trained technicians conducted the water quality assessments or the analyses were undertaken by laboratory technicians?
External review	Was the study subject to peer review or external review prior to publication?

### Analysis

Because of the extent of heterogeneity between studies, we chose to plot cumulative density functions (CDFs) of the proportion of samples with detected (>1 per 100 ml) and high (>100 per 100 ml) FIB in each study to compare water source types between studies. This approach has been used in a systematic review of prevalence of schizophrenia [40]. CDFs are used to qualitatively assess the proportion of studies reporting frequent and high levels of microbial contamination. Measures of central tendency from studies were not included in the meta-analysis because of limited reporting of measures of dispersion, inadequate explanation of the handling of censored data, and the difficulty in reconciling diverse reported measures of central tendency (e.g., geometric versus arithmetic mean) [41].

Random effects meta-regression was used to investigate risk factors and settings where fecal contamination is most common and other possible explanations for the observed heterogeneity between studies [42]. A logit transformation is recommended for the analysis of proportions [43] and was applied to both the proportion of samples with detectable (>1 per 100 ml) and high (>100 per 100 ml) levels of FIB. The *metareg* function in Stata was used after a continuity correction of ±0.5 where the proportion of samples positive was zero or one [44], and we estimated the within study variance for each proportion as the reciprocal of the binomial variance [45]. Subgroup analysis included variables defined *a priori* (including water source type, rural versus urban, and income-level) and defined *a posteriori* (for example if piped water had been treated prior to distribution). We separately evaluated piped and other improved sources for those variables reaching significance at the 5% level in bivariate analysis for all source types.

Studies that included both improved and unimproved sources were then combined using meta-analysis with the odds ratio (OR) as the effect measure. We calculated a pooled estimate of the protective effect of an improved source and corresponding confidence intervals using the *metan* function in Stata. We then assessed the influence of small study bias by the funnel plot method and performed an Egger's test using a normal likelihood approximation. The extent of heterogeneity in protective effect was determined using Higgins I^2^ and corresponding confidence intervals were calculated [42]. Calculations were performed in Stata 13SE.

## Results

### Search Results

**Figure 2:**
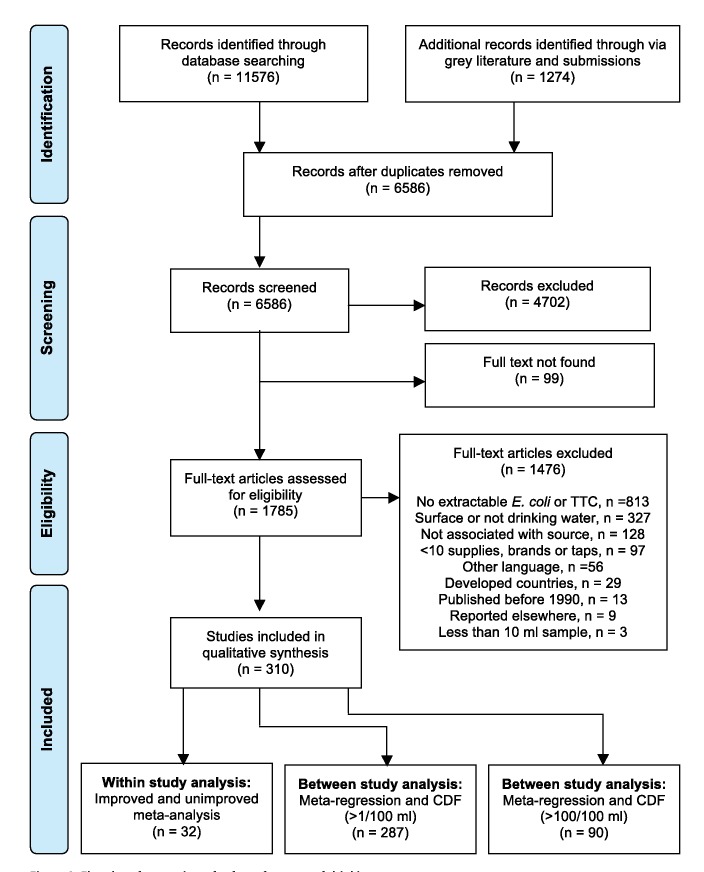
Flowchart for a review of safety of sources of drinking-water.

As shown in Figure 2, in total, 6,586 reports were identified through database searches. A further 1,274 reports were identified from grey literature and correspondence with experts. Most studies were excluded because they did not test water that was clearly used for drinking, did not associate results with a water source type, or did not include enough different water sources or in the case of packaged water, brands. Studies often did not provide an adequate description of the water sources to allow them to be matched to the JMP source categories; this limitation was particularly the case for ground water sources. For example, several studies reported results for “hand pumps” (a description of the technology above ground) but did not provide details about well construction. Although these may often be boreholes, hand pump conversions are also applied to dug wells. Other studies simply described water sources as “wells” or “springs.” Some studies provided details that are not captured in the JMP classification, such as whether water from a piped supply had been treated. Full texts could not be obtained for 99 potentially relevant reports, many of which were conference presentations and most of which were identified from bibliographies. The remaining 310 reports [6],[24],[46]–[353] were incorporated in our review and provide information on 96,737 water samples. The total number of studies is higher (319) due to a small number of multi-country reports. On average each study provides information on 1.7 water source types, resulting in a database with 555 datasets (Dataset S1).

### Study Characteristics

Characteristics of included studies are summarized in Table 3. The review is dominated by cross-sectional studies (*n* = 241, 75%) with fewer longitudinal surveys (*n* = 39, 12%). Authors report selecting sources or households at random in a minority of studies (*n* = 68, 21%); most of these studies selected sources randomly within a region or community rather than at national level. The main exceptions were the Rapid Assessment of Drinking-Water Quality (RADWQ) studies commissioned by WHO and UNICEF, of which five have been published [64],[65],[164],[281],[322] and a repeated cross-sectional study in Peru for which only the total coliform results have previously been reported but for which we were able to secure *E. coli* data [227].

**Table 3:** Characteristics of included studies.

Characteristic	Studies	Datasets	Samples
	Number (%)	Number (%)	Number (%)
**Setting**			
Urban	146 (46)	227 (41)	30,038 (31)
Rural	130 (41)	243 (44)	34,850 (36)
Both urban and rural	41 (13)	83 (15)	31,767 (33)
Unclassified setting	2 (1)	2 (0)	82 (0)
Emergencies	13 (4)	26 (5)	2,897 (3)
Non-household	17 (5)	21 (4)	2,121 (2)
**Point of sampling**			
Stored water	50 (15)	74 (13)	19,965 (21)
Directly from source	293 (92)	481 (87)	76,772 (79)
**Water supply**			
*Improved*	209 (65)	273 (49)	56, 268 (58)
Piped	118 (37)	119 (21)	32,348 (33)
Borehole	83 (26)	83 (15)	11,452 (12)
Protected dug well	36 (11)	36 (6)	8,697 (9)
Protected spring	11 (3)	11 (2)	978 (1)
Rainwater	25 (8)	25 (5)	2,793 (3)
*Unimproved*	62 (19)	71 (13)	5,594 (6)
Unprotected dug well	49 (15)	49 (9)	4,577 (5)
Unprotected spring	16 (5)	16 (3)	810 (1)
Tanker truck	6 (2)	6 (1)	207 (0)
*Unclassified*	167 (53)	213 (38)	35,087 (36)
Sachet	15 (5)	15 (3)	1,305 (1)
Bottled	35 (11)	35 (6)	2,339 (2)
Dug well	49 (15)	49 (9)	4,577 (5)
Spring	16 (5)	16 (3)	810 (1)
**Design**			
Randomized	68 (21)	131 (24)	31,210 (32)
Representative	74 (23)	148 (27)	37,614 (39)
Cohort or case control	5 (2)	15 (3)	4,114 (4)
Intervention	22 (7)	47 (8)	9,799 (10)
Cross-sectional survey	241 (75)	404 (73)	48,559 (50)
Longitudinal survey	39 (12)	66 (12)	32,302 (33)
Diagnostic	12 (4)	23 (4)	1,963 (2)
**Parameter**			
*E. coli*	152 (48)	270 (49)	32,298 (33)
TTC only	167 (52)	285 (51)	64,439 (67)
**Language**			
English	276 (86)	502 (90)	81,349 (84)
Spanish	6 (2)	8 (1)	3,024 (3)
Portuguese	24 (8)	29 (5)	9,146 (9)
French	4 (1)	5 (1)	187 (0)
Chinese	9 (3)	11 (2)	3,031 (3)
**Reporting**			
Presence/absence of FIB	287 (90)	499 (90)	90,056 (93)
Microbial risk classification	90 (28)	165 (30)	23,953 (25)
Mean FIB	80 (25)	136 (25)	15,530 (16)
Geometric mean FIB	34 (11)	68 (12)	11,797 (12)
Range of FIB	74 (23)	108 (19)	9,407 (10)
Standard deviation of FIB	21 (7)	38 (7)	4,417 (5)
Sanitary risk	44 (14)	82 (15)	15,808 (16)
WHO sanitary risk	12 (4)	31 (6)	9,160 (9)
Sanitary risk classification	17 (5)	44 (8)	10,667 (11)
**Sample Size** a			
Small (*n* = 10–30)	NA	192 (35)	3,711 (4)
Medium (*n* = 31–100)	NA	187 (34)	11,615 (12)
Large (*n* = 101–6,021)	NA	176 (32)	81,411 (84)
**Quality** b			
Low (1–5)	113 (36)	199 (36)	27,892 (29)
Medium (6–7)	94 (29)	142 (26)	16,980 (17)
High (8–13)	112 (35)	214 (39)	51,865 (54)
Total	319 (100)	555 (100)	96,737 (100)

a Terciles by datasets.

b Terciles by study.

NA, not applicable.

**Figure 3:**
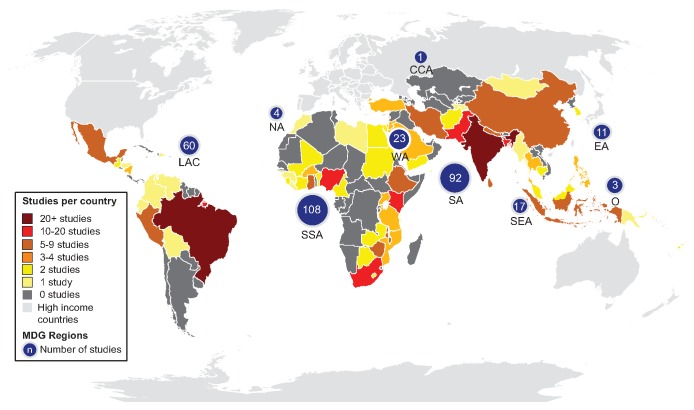
Map of study locations.

Study quality varied greatly spanning from a quality score of 1 to 12 and with an interquartile range of 5 to 8 (Figure S1). Whereas most studies described the analytical method used to detect *E. coli* or TTC (80%), how water sources were selected (67%), and the setting in which the study took place (86%), fewer specified quality control procedures (15%), met the basic sample handling criteria (25%), used trained technicians to conduct the water quality tests (15%), or arranged testing in an accredited laboratory (12%) (Figure S2). Most studies were from sub-Saharan Africa, southern Asia, or Latin America and the Caribbean (Figure 3). The majority of included studies investigated water quality at the source. Studies reporting on the quality of water stored in households by provenance were less common (*n* = 49), and few of these compare quality of stored water with that of the associated source (*n* = 26). Several studies took place during or after emergencies [97],[201] and natural hazards, including cyclones [235], floods [78],[208], droughts [341], and tsunamis [130],[147],[331]. Non-household settings such as schools and health facilities were addressed in a small number of studies (*n* = 17). Few studies separately report water quality information from slum or peri-urban settings (*n* = 7).

### Qualitative Synthesis

In Figures S3 and S4 levels of microbial contamination are shown using the FIB level classification (<1, 1–10, 10–100, and >100 FIB per 100 ml), grouped by type of improved water source. These results are broadly in agreement with a comparison using measures of central tendency (Figure S5) and show great variability in the likelihood and extent of contamination between studies and source types.

Large studies with random sampling demonstrate marked differences in water quality between countries; for example less than 0.01% of samples from utility piped supplies in Jordan [281] were found to contain TTC compared with 9% to 23% of utility piped supplies in the other four RADWQ countries [64],[65],[164],[322]. Only one national randomized study differentiated between rural and urban areas; the proportion of samples from piped supplies containing *E. coli* was found to be substantially higher in rural (61%, *n* = 101) than urban (37%, *n* = 1470) areas in Peru [227].

In comparison to microbial testing, sanitary inspections are less widely practiced or data are rarely published. Sanitary inspection procedures vary considerably and are usually adapted to the local context; of the 44 studies reporting sanitary inspections only 12 used standardized WHO forms. In Figure S6 the sanitary risk levels as reported in nine studies are compared with the proportion of samples containing FIB and suggest that there is no strong association between these two measures.

### Between Studies Analysis: CDF and Meta-regression

The number of studies reporting high proportions of samples contaminated or high levels of FIB is lower for improved sources as can be seen in Figure S7. Yet, in 38% of 191 studies reporting the quality of improved sources, at least a quarter of samples exceeded recommended levels of FIB. Figure S8 shows CDFs by source type with similar patterns to those from the FIB level classification.

Results of the meta-regression are shown in Table 4. We find that country income-level is a significant determinant of water quality and the odds of contamination are 2.37 times (95% CI 1.52–3.72 [*p* = 0.001]; Table 4) higher in low-income countries compared with wealthier countries. However this result is not significant when separately considering piped and other improved sources (Tables S1 and S2).

**Table 4:** Between studies meta-regression.

Variables	Proportion of Samples >1 FIB per 100 ml			Proportion of Samples >100 FIB per 100 ml		
	Obs.	OR [95% CI]	*p*-Value	Obs.	OR [95% CI]	*p*-Value
***Source type***						
Improved vs. unimproved	291	0.14 [0.08–0.25]	<0.001	87	0.13 [0.05–0.33]	<0.001
Piped vs. other improved	239	0.53 [0.32–0.89]	0.017	68	0.47 [0.18–1.20]	0.11
Protected vs. unprotected groundwater	90	0.26 [0.11–0.60]	0.002	31	0.37 [0.09–1.52]	0.16
Treated piped vs. untreated piped^a^	69	0.07 [0.02–0.27]	<0.001	18	0.10 [0.01–0.72]	0.025
Stored vs. source	474	2.09 [1.16–3.78]	0.015	140	1.85 [0.68–5.04]	0.23
***Setting***						
Low-income vs. other	414	2.37 [1.52–3.71]	<0.001	122	1.30 [0.59–2.86]	0.52
Rural vs. urban	344	2.37 [1.47–3.81]	<0.001	96	1.18 [0.49–2.83]	0.71
***Study characteristics***						
Thermotolerant vs. *E. coli*	417	1.08 [0.70–1.67]	0.72	122	0.99 [0.45–2.19]	0.98
Publication year	415	0.96 [0.93–1.00]	0.029	122	0.96 [0.91–1.02]	0.16
Random vs. non-random selection	417	0.92 [0.53–1.57]	0.75	122	0.60 [0.25–1.44]	0.25
High quality vs. lower quality^b^	417	0.90 [0.54–1.49]	0.68	122	0.51 [0.21–1.23]	0.13
Longitudinal vs. cross-sectional	372	1.00 [0.58–1.73]	0.99	116	1.05 [0.40–2.73]	0.93
Wet vs. dry	51	0.99 [0.32–3.10]	0.99	22	0.93 [0.09–9.34]	0.95
***Reporting format***						
Measure of central tendency	417	2.31 [1.45–3.69]	<0.001	122	1.22 [0.55–2.68]	0.62
Microbial risk classification	417	2.30 [1.45–3.67]	<0.001	—	—	—

With the exception of stored versus source, we restricted the analysis to source water samples. We excluded emergencies from the meta-regression.

a Post hoc analysis.

b Top tercile of studies versus bottom two terciles.

Obs.,number of observations.

Meta-regression showed a substantial difference in the proportion of samples containing FIB between urban and rural areas (OR = 2.37 [95% CI 1.47–3.81], *p*<0.001). There is weak evidence to suggest that piped supplies are more likely to be contaminated in rural areas (OR = 2.4 [95% CI 0.98–5.92], *p* = 0.054; Table S1), but no evidence of differences for all other improved source types (OR = 1.19 [95% CI 0.52–2.72], *p* = 0.67; Table S2).

Protection of groundwater (OR = 0.26 [95% CI 0.11–0.60]; *p* = 0.002; Table 3) and treatment of piped water (OR = 0.07 [95% CI 0.02–0.27], *p*<0.001; Table 3) were both strongly related to better water quality. Contamination of stored water was more likely than water at the source (OR = 2.09 [95% CI 1.16–3.78], *p* = 0.015; Table 3), including for piped supplies (OR = 2.35 [95% CI 1.08–5.12], *p* = 0.032; Table S1).

### Within Studies Analysis: Meta-analysis

**Figure 4:**
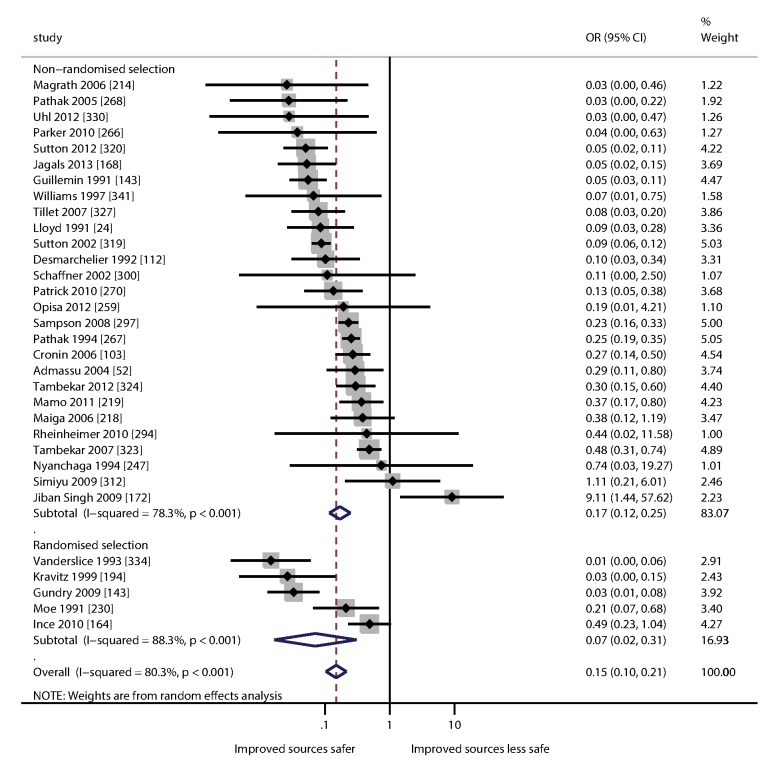
Forest plot of the odds of fecal contamination for improved and unimproved sources.

Figure 4 is a forest plot showing the ORs of contamination for improved sources compared to unimproved sources from eligible studies. Meta-analysis of randomized and non-randomized studies showed that improved sources are less likely to be contaminated (pooled OR = 0.15 [0.10–0.21]; Figure 4); the protective effect was found to be greater in randomized studies and none of the randomized studies found contamination to be more frequent in improved sources. Heterogeneity was relatively high (I^2^ = 80.3% [95% CI 72.9–85.6) indicating that the protective effect varies considerably between settings. The OR for a small number of studies was greater than one, suggesting that in some settings improved sources may not always be less likely to contain FIB than the unimproved alternative.

### Assessment of Bias

Egger's test found no evidence of small study effects for the meta-analysis of improved versus unimproved sources (*p* = 0.64; Figure S9). Meta-regression did not detect evidence of bias due to study design, lack of randomization, study quality, or season (“wet” or “dry”). As expected, we find that levels of FIB (risk classification or measures of central tendency) were more likely to be reported in studies where water was more contaminated with FIB (Tables 4, S1, and S2) and therefore may constrain comparisons between studies. Publication year was also related to the proportion of samples containing FIB (OR = 0.96 [95% CI 0.93–1.00]; *p* = 0.029; Table 4), but this was not significant when separately considering piped and other improved sources (Tables S1 and S2). Studies testing the same sources in different seasons report considerable variation in microbial water quality (Table S3).

## Discussion

### Safety of Improved Sources

We demonstrate that water from improved sources is less likely to contain FIB than unimproved sources. Using meta-analysis we compare water from unimproved sources and improved sources within the same study and found improved sources were less likely to contain FIB (OR = 0.15 [95% CI 0.10–0.21]) (Figure 4), with the greatest protective effects in studies where selection of water sources was randomized. Comparison between studies of improved versus unimproved sources yielded an OR of 0.14 (95% CI 0.08–0.25) for the presence of FIB and 0.13 (95% CI 0.05–0.34) for samples exceeding 100 FIB per 100 ml (Table 4). Cumulative density plots show markedly lower contamination for improved sources relative to unimproved sources.

While improved sources clearly offer a greater degree of protection compared to unimproved sources, they are not all nor consistently safe [354]. In particular, protected dug wells were rarely free of fecal contamination and it is not uncommon for these sources to contain high levels of FIB. High levels of contamination were occasionally reported for boreholes and piped water, which are typically perceived as high quality and lower risk. Risk factors for microbial contamination of piped supplies include intermittency [198] and inadequate chlorination [20],[355]. For boreholes and dug wells, the reasons for fecal contamination can be more difficult to ascertain owing to the possibility of aquifer contamination and/or inadequate sanitary completion. In many cases contamination is associated with poor hygiene and inadequate sanitation, but specific risks can be readily identified through sanitary inspection of the water source and its surroundings and may in part explain the heterogeneity in FIB concentrations observed for a given source type.

Studies included in this review show great variability in water quality for water sources of a given type, suggesting considerable scope for reducing exposure to fecal contamination through systematic management of water safety. Microbial water quality displays substantial heterogeneity between studies and we found a high I^2^ when comparing improved and unimproved sources from the same study (I^2^ = 80.3% [72.9–85.6]; Figure 4).

Across all studies, we find a higher risk of contamination in rural areas compared to urban areas (OR = 2.37 [1.47–3.81], *p*<0.001; Table 4) and a higher risk of contamination in low-income countries (OR = 2.37 [1.52–3.71], *p*<0.001; Table 4). Higher risk in rural areas is consistent with a recent multi-country study of over 25,000 hand pumps, which found greater risk of non-functioning water sources in areas distant from district centers [356].

There is some evidence to suggest that overall water quality has gradually improved over time. Publication year was associated with the proportion of samples containing FIB (OR 0.96 [0.93–1.00], *p* = 0.023; Table 4); this may reflect a progressive trend towards greater use of source types associated with less contamination and potentially a lessening of population-level exposure.

We found only limited published data on sanitary risk, suggesting that sanitary risk inspection techniques are not widely used and/or reported, despite being well-established in national and international drinking-water guidelines [12]. Studies report sanitary risks in some sources that are not found to contain detectable FIB, indicating that infrequent monitoring does not provide assurance of water safety. Conversely, low sanitary risk scores are reported for water sources that contain FIB, indicating that sanitary inspections alone do not capture water safety fully. Although individual studies report different levels of sanitary risk, the data are too few to draw general conclusions. Moreover, the use of different questions for each source type and their equal weighting limits the comparability of sanitary risk scores. While a simple prevalence of factor scoring is unlikely to be appropriate in more complex systems, there has been progress in developing scoring for quality of water safety plans and higher scoring utilities have been linked to both improved water quality and health outcomes [357].

### Limitations of the Review

At the outcome level, our principal analyses are based on the proportion of samples detecting FIB rather than compliance to health guidelines over the course of a year. These prevalence measures overestimate compliance to health guidelines and national standards that require minimum sampling frequencies. Furthermore, where the sample volume is less than the recommended 100 ml [12], contamination is less likely to be detected and will be detected less frequently.

At the study level, our review was limited by infrequent reporting of a consistent measure of central tendency (or of individual sample data), sanitary risk inspections, and stored water quality. In analysis, we combine studies that used diverse sample handling and microbiological analytical methods and these factors may account for some of the variability in reported water quality. The majority of the included studies were cross-sectional and do not provide information on temporal variability in water quality. Few studies achieved robust random selection of water sources, and few received high scores for study quality (14% with >9 out of 13; Figure S1) with description of quality control procedures, meeting handling criteria, and statement of season(s) of sampling most frequently omitted quality factors. Many studies, particularly of groundwaters, were excluded as we could not match water source types or determine whether they were “improved.”

At the review level, we may not have identified all studies that meet the inclusion criteria. To capture additional studies would have required the screening of tens of thousands of records, as we were unable to identify more specific search terms. Two sources of water quality information that could be used in future studies and monitoring: regulatory surveillance and utility quality control data are likely to be extensive and not well represented as they may not be published and publicly available. Publicly available data from these sources rarely matched our inclusion criteria, usually because of failure to report sample sizes or associate water quality with source type. We identified few studies in languages other than English despite conducting searches in four other languages, and several regions are underrepresented (Figure 3) including Caucasus and Central Asia and Oceania for which studies may be available in other languages. Since few studies separately report water quality in slums, we combined studies of slum and peri-urban populations with those taking place in formal urban areas and we were therefore unable to investigate intra-urban disparities [7]. There may be a small number of errors in the database; in the 10% of English language studies independent extraction <0.5% errors were identified.

There are two sources of bias that will tend to cause overestimation of the safety of improved sources. Firstly, for many source types, water (including unreliable piped systems, public standpipes, and wells) is collected at the source, carried to, and stored in the household—affording multiple opportunities for contamination—such that final water quality is often worse than in the associated source [34]. Few studies identified the source type of origin of stored water; those that did supported the suggestion that stored water is more frequently contaminated and contaminated at higher levels. Data interpretation is confounded because samples are not paired [204] or are temporally displaced. Despite the potential to improve matters, evidence for the impact of household water treatment on stored water quality is inconsistent [295],[358]. Secondly, most studies were cross-sectional. One-off or infrequent sampling overestimates safety by missing seasonal [359],[360] or sporadic contamination and longitudinal studies suggest seasonal effects can be substantial (Table S3).

### Monitoring Implications: Improving on Improved

There is a widely perceived hierarchy for water source desirability, typically with piped-to-household-tap as the ideal. Such general hierarchies combine many aspects of water service and their value, including quantity, affordability, accessibility, and reliability or continuity of service as well as safety [361]. It has been argued that a more graduated approach to monitoring than the “improved”/“unimproved” dichotomy is required [24],[362]. These concepts were reflected in initial JMP working group proposals that called for the monitoring of “basic” and “intermediate” service levels representing improvements in quality, continuity of supply, and accessibility [30]. Such monitoring could contribute to assessing progressive realization of the Human Right to Water and Sanitation [363] and to encourage improvements in service delivery, including improvements within water source-type categories.

A variety of indicators could be used to improve global estimates of water safety. Account of safety could be enhanced by ranking or scoring improved source types according to the proportion of sources showing fecal contamination (in descending order): piped (treated), boreholes, protected springs, rainwater, piped (untreated), and protected dug wells. Although based on *a posteriori* analysis, we find that untreated piped supplies are much more likely to be contaminated than treated piped supplies (OR = 0.07 [95% CI 0.02–0.27], *p*<0.001; Table 4) and may usefully be considered separately. This ranking could be refined as more data become available but may have unintended consequences such as disincentivising improvements within each source type and could discourage the use of some source types in regions where these may in fact provide comparatively safe water. We find that bottled and sachet water are typically high quality, but their environmental sustainability has been questioned [318]. They are excluded from global progress efforts not for reasons of quality but because they provide insufficient quantity for domestic uses, such as cooking and hygiene, other than direct consumption.

Adjustment of the improved source indicator for country-specific source-type compliance with microbial water quality guidelines would capture the substantial heterogeneity both between and within countries, highlighting disparities in the use of safe drinking-water [364]. It would provide a more robust and consistent means of assessing safety than the use of source-type classifications alone and would enable improvements in quality to be reflected in monitoring. This review indicates that such an adjustment would have a large (downward) impact on international estimates of the number of people using safe drinking-water, in agreement with previous estimates based on five studies [8],[9].

Prevalent FIB are imperfect indices of water safety [26] and health risk. They are known to be more sensitive to chlorine than some important waterborne pathogens such as cryptosporidium [12], and can persist or multiply in some tropical waters [365]. These factors and the fact of temporal variability in water quality suggest that it would be strongly preferable to combine periodic measurement of water quality with assessment of sanitary status through sanitary inspection or water safety plans [29] in assessing water safety. These approaches can serve to highlight the condition, operation, and maintenance of water sources and provide a more complete picture than access to infrastructure alone. Such approaches would benefit from standardization to ensure comparability. However, adjustment for the proportion of samples containing FIB would not account for all hazards to health, including the two major chemical hazards: fluoride [366],[367] and arsenic [368]. The JMP has outlined an approach that could be taken that combines a hierarchy of measures of water quality with sanitary risk or management data [22].

Cost-effectiveness arguments suggest basing future monitoring efforts on both regulatory and utility data and building monitoring capacity especially in peri-urban and rural areas of low-income countries. Special initiatives such as dedicated water safety surveys (e.g., RADWQ) and integration of water quality testing in household surveys are likely to play an interim role and will assist in filling gaps in the available data [22].

### Implications for Public Health Policy

Our review provides strong evidence that by equating “improved” with “safe,” the number of people with access to a safe water source has been greatly overstated, and suggests that a large number and proportion of the world's population use unsafe water. We analyze the implications following a framework of health sector functions in environmental health [369] and highlight key implications for policy in Box 1.

**Box 1. Key Implications for Policy**

- Fecal contamination of drinking-water is widespread globally, especially in low-income countries and rural areas, and affects many improved sources.
- The Global Burden of Disease 2010 study may greatly underestimate diarrheal disease burden by assuming zero risk from improved sources.
- Adjustment of safe drinking-water coverage estimates for water quality and ideally sanitary risk would highlight disparities and enable improvements in quality to be reflected in monitoring.
- Piped water is not a panacea: high levels of contamination have been reported in a range of settings and water stored in the household, often motivated by an intermittent or distant source, is more likely to be contaminated, especially in rural areas.
- Quality and sanitary risks are heterogeneous indicating that it is possible to substantially enhance safety and reduce exposure through incremental improvements in service.
- Greater use should be made of sanitary inspections as these provide a complementary means of assessing safety and are able to identify corrective actions to prevent contamination.
- Studies of microbial contamination and sanitary risk could be improved by adhering to higher standards, including those outlined in our quality criteria.

#### Policy makers

Health policy makers framing post-2015 goals for achieving universal health coverage and reducing the global burden of disease need to ensure that targets and indicators go beyond health care services and address underlying determinants of health including progressive improvements in access to safe drinking-water, sanitation, and hygiene services. Adequate quantities of safe water at home are essential for good health [370] and, together with improvements in sanitation and hygiene, considered one of the more cost-effective interventions to protect and improve public health [371].

Through the process of implementing the MDGs, great strides have been made in increasing coverage of improved water sources although an estimated 783 million people still lack an improved source, most living in sub-Saharan Africa and Southeast Asia [5]. This review confirms that there are also pronounced disparities in access to “safe” water between and within countries. The Human Right to Water and Sanitation calls for progressive reduction in inequalities, and public health considerations suggest that reducing exposure among the most vulnerable, including the poor, undernourished, and immunocompromised, is a key public health concern. Health policy makers therefore have an important role to play in advocating for health protecting policies in other sectors, including those by actors concerned with water supply services.

#### Standard setting for water quality

The health sector plays a substantive role in drinking-water quality standard setting in many countries; this is logical since the underlying rationale is based on health concerns. The evidence presented here suggests that failures in water safety are frequent in LMICs, and that effective standard setting would combine outcomes measures (such as the measurement of FIB) with the verification of preventive or protective measures through sanitary inspection, water safety plans, or similar approaches. Future standard setting should take account of inequalities in access so as to direct efforts to those most affected and be informed by the availability of effective interventions.

#### Surveillance

The health sector also plays an important role in environmental health including drinking-water quality surveillance in many countries. However in many LMICs surveillance of water quality is limited outside large consolidated urban centers and enforcement of guidelines can be weak [372]. Surveillance is typically weakest in rural areas where levels of access to “improved sources” are lowest and the likelihood of contamination is greatest. The lack of disaggregated data for peri-urban and urban slum areas is also a problem in many countries. To-date the focus of public health policy, targets, and monitoring has been a household's primary source of drinking-water [373], but there is growing concern over inadequate water, sanitation, and hygiene services in non-household settings, such as schools and health care facilities [30],[373]. A public health perspective suggests that health sector surveillance should focus particularly on these settings where the risk of exposure is high.

#### Health care settings

Ensuring adequate environmental health in health care settings is a key responsibility of the health profession [374], but in many countries access to water, sanitation, and hygiene in health care facilities remains inadequate. While data were few, those studies that addressed water safety in health care settings documented water safety deficiencies [142],[235]. Furthermore, nationally representative surveys of health care facilities in Uganda and Rwanda found two-thirds of health care facilities to lack an improved water source [375],[376]. There are also opportunities to better protect and improve health through incorporation of water safety components in health programs such as those focused on specific diseases [377] or sensitive life stages such as maternal and child health [378]. Given the vulnerability of the populations using them and the potential for health facilities to serve as models for the wider community, the health sector has both a duty of care and an opportunity to advance health through better management of water safety in its own facilities.

#### Outbreak investigation

Despite clear evidence of its prevalence, the global burden of disease attributable to fecal contamination of drinking-water remains poorly understood. Data on outbreaks of waterborne disease [19] and of the impact of interventions to improve water quality on endemic disease [15],[16] provide evidence of the importance of fecal contamination. The associated studies frequently provide only weak insight into causal factors that might otherwise contribute to improved preventive action. There is an opportunity to enhance current outbreak investigation, advancing its role from one of curtailment to general prevention, thereby improving the ability to retrieve information that can be generalized for future prevention.

#### Waterborne disease burden

Study of the national and global burden of disease provides an opportunity to enhance public health protection and increase cost-effective action by focusing efforts on disease burdens and risk factors of greatest significance. The recent Global Burden of Disease study [379] based its estimates on the assumption of zero risk for those supplied by improved drinking-water sources and no additional benefit of a piped supply on premises [354]. The findings of this review indicate that fecal contamination of drinking-water is widespread, particularly in rural areas and low-income countries. Some improved source types, especially protected dug wells and protected springs, are frequently and sometimes highly contaminated. Contamination reported in piped supplies in especially rural but also urban areas is concerning given that these serve the majority (63%; Table 1) of the world's population and the use of this source type is expanding rapidly in many countries, especially in China [380]. In assuming improved sources are safe [379], current estimates may greatly underestimate waterborne disease burden and this gap would be expected to grow as improved source coverage increases.

### Implications for Research

We have applied analytical tools usually associated with the medical sciences (meta-analysis of prevalence) to the study of environmental contaminants. There are differences in the underlying data that merit highlighting and limit the transferability of these techniques. Firstly, robust sampling frames are usually available for the selection of households (e.g., from national statistical offices), but random selection of water sources is more challenging. Studies such as RADWQ that adapted stratified cluster sampling techniques used in household surveys to address this problem have been subject to methodological criticism [381]. Future directions to achieve representative samples could include the use of water point mapping [382] or satellite imagery to create lists of water points but both are relatively complex and approaches based on population may be more feasible. Secondly, whereas epidemiological studies seek to measure outcome variables such as the number of events at a given point in time (“point prevalence”) or the rate at which they occur (“incidence”), environmental studies often seek to determine whether a threshold condition of safety has been exceeded (“compliance”). Even brief failures in safety may negate much of the potential health benefits of otherwise safe water [383],[384]. As a consequence, assessments of safety can be particularly susceptible to the frequency of monitoring and temporal representativity can be as important as spatial or population-based randomization.

In order to assess levels of compliance with regulatory standards or international guidelines based on infrequent surveys or limited data, research is required to understand the effect of repeated sampling of both source and stored water that results from variability over time (e.g., seasonality) and replicate sampling (sequential testing). Given their potential to inform assessments of safety, there is also a need to improve understanding of the relative importance of sanitary risks and their temporal variation for which very limited data are currently available. There is unlikely to be a simple correlation between sanitary risks and microbial contamination [103],[209] but the predictive value of sanitary risks may be much higher when accounting for compliance over time because some infrastructure failures will only lead to contamination in the presence of a co-factor such as rainfall. There is also a need to better understand the role of water collection and storage on microbial contamination and the associated risk to health.

We find strong evidence of differences in water quality between rural and urban areas, including for piped supplies. Further work is needed to characterize intra-urban differences; we encourage randomized water quality surveys to include slum or peri-urban populations as part of the sampling frame. Finally, the approaches taken in this review could be extended to other drinking-water contaminants, such as arsenic and fluoride.

## Conclusions

Fecal contamination of drinking-water in LMICs is widespread. We demonstrate that improved sources are in general safer than unimproved sources of drinking-water, but they are not universally nor consistently free of fecal contamination. In 38% of 191 studies at least a quarter of samples from improved sources exceeded WHO recommended levels of FIB. By equating “use of an improved source” with “safe,” international estimates greatly overstate access to safe drinking-water. Substantial differences are observed in the presence and levels of contamination between countries, between urban and rural regions, and between water source types. Infrequent measurements of water quality alone tend to overestimate safety and so an improved future strategy would combine sanitary status with water quality measurements.

## Supporting Information

Figure S1 **Study quality rating for 319 studies.**
(EPS)

Figure S2 **Frequency of 13 quality criteria being met by 319 included studies.**
(EPS)

Figure S3 **Fecal indicator bacteria level classification for improved sources by source type and study.** Included studies are those for which a FIB level classification was reported or could be calculated. Sample size in curved parentheses. Reference number in square brackets. Source types are: BH, borehole; PWS, piped water supply; PS, protected spring; PDW, protected dug well; RWH, rainwater harvesting.
(EPS)

Figure S4 **Fecal indicator bacteria level classification for unimproved and unclassified sources by source type and study.** Included studies are those for which a FIB level classification was reported or could be calculated. Sample size in curved parentheses. Reference number in square brackets. Source types are: UDW, unprotected dug well; US, unprotected spring; TT, tanker truck; B, bottled; S, sachet. NDW, dug well (unclassified); NS, springs (unclassified).
(EPS)

Figure S5 **Measures of central tendency reported by included studies, by source type.** Size of circles proportional to number of water samples evaluated.
(EPS)

Figure S6 **Comparison of sanitary risk levels and proportion of samples containing fecal indicator bacteria in studies using WHO standardized inspection forms.**
(EPS)

Figure S7 **Cumulative density functions for the proportion of samples in each study with detectable (>1 per 100 ml) and high (>100 per 100 ml)** ***E. coli*** **or TTC, by improved and unimproved source.**
(EPS)

Figure S8 **Cumulative density function of the proportion of samples containing fecal indicator bacteria in each study for improved (left) and unimproved (right) sources by type.**
(EPS)

Figure S9 **Funnel plot for the odds ratio comparing the safety of improved and unimproved sources in a given study.**
(EPS)

Table S1 **Between studies meta-regression for piped supplies.**
(DOCX)

Table S2 **Between studies meta-regression for other improved sources.**
(DOCX)

Table S3 **Variation in microbial safety during the year, findings of included studies for selected source types.**
(DOCX)

Alternative Language Abstract S1 **Mandarin Chinese translation of the abstract by Hong Yang.**
(DOCX)

Dataset S1 **Database of included water quality studies.**
(XLSX)

Protocol S1 **Systematic review protocol.**
(DOCX)

Text S1 **PRISMA checklist of items to include when reporting a systematic review or meta-analysis.**
(DOC)

## Abbreviations

CDF: cumulative density function
FIB: fecal indicator bacteria
JMP: Joint Monitoring Programme
LMICs: low- and middle-income countries
MDG: Millennium Development Goal
OR: odds ratio
RADWQ: Rapid Assessment of Drinking-Water Quality
TTC: thermotolerant coliform
