# Supplementary material for: Fecal Contamination of Drinking-Water in Low- and Middle-Income Countries: A Systematic Review and Meta-Analysis
Source: PLoS Med. 2014 May 6;11(5):e1001644. doi: 10.1371/journal.pmed.1001644 (PMC4011876; doi:10.1371/journal.pmed.1001644)
Supplement: Table S1 — Between studies meta-regression for piped supplies. (DOCX) [file pmed.1001644.s010.docx]

**Table S1: Between studies meta-regression for piped supplies**

| **Variables** | **Proportion of samples >1 FIB per 100 ml** | | | **Proportion of samples >100 FIB per 100 ml** | | |
| --- | --- | --- | --- | --- | --- | --- |
|  | **Obs.** | **OR** [95% CI] | **p-value** | **Obs.** | **OR** [95% CI] | **p-value** |
| ***Setting*** |  |  |  |  |  |  |
| Low-income vs other | 100 | 1.21 [0.44-3.31] | 0.706 | 22 | 1.12 [0.17-7.55] | 0.903 |
| Rural vs urban | 85 | 2.41 [0.98-5.92] | 0.054 | 16 | 2.64 [0.46-15.13] | 0.253 |
| ***Sampling location*** |  |  |  |  |  |  |
| Stored vs source | 129 | 2.35 [1.08-5.12] | **0.032** | 30 | 3.99 [0.96-16.58] | 0.056 |
| ***Study characteristics*** |  |  |  |  |  |  |
| Publication Year | 100 | 0.98 [0.92-1.05] | 0.640 | 22 | 0.93 [0.79-1.09] | 0.346 |
| ***Reporting format*** |  |  |  |  |  |  |
| Measure of central tendency | 101 | 1.98 [0.78-5.05] | 0.151 | 22 | 1.73 [0.23-13.02] | 0.576 |
| Microbial risk classification | 101 | 1.08 [0.41-2.83] | 0.875 | - | - | - |
