# Supplementary material for: Fecal Contamination of Drinking-Water in Low- and Middle-Income Countries: A Systematic Review and Meta-Analysis
Source: PLoS Med. 2014 May 6;11(5):e1001644. doi: 10.1371/journal.pmed.1001644 (PMC4011876; doi:10.1371/journal.pmed.1001644)
Supplement: Table S2 — Between studies meta-regression for other improved sources. (DOCX) [file pmed.1001644.s011.docx]

**Table S2: Between studies meta-regression for other improved sources**

| **Variables** | **Proportion of samples >1 per 100 ml** | | | **Proportion of samples >100 per 100 ml** | | |
| --- | --- | --- | --- | --- | --- | --- |
|  | **Obs.** | **OR** [95% CI] | **p-value** | **Obs.** | **OR** [95% CI] | **p-value** |
| ***Setting*** |  |  |  |  |  |  |
| Low-income vs other | 138 | 1.21 [0.63-2.33] | 0.565 | 46 | 1.71 [0.61-4.78] | 0.300 |
| Rural vs urban | 111 | 1.19 [0.52-2.72] | 0.671 | 33 | 0.70 [0.18-2.7] | 0.597 |
| ***Sampling location*** |  |  |  |  |  |  |
| Stored vs source | 183 | 2.29 [1.11-4.72] | **0.026** | 53 | 0.80 [0.26-2.46] | 0.694 |
| ***Study characteristics*** |  |  |  |  |  |  |
| Publication Year | 137 | 0.99 [0.94-1.04] | 0.710 | 46 | 0.99 [0.92-1.07] | 0.837 |
| ***Reporting format*** |  |  |  |  |  |  |
| Measure of central tendency | 138 | 1.50 [0.77-2.93] | 0.228 | 46 | 0.67 [0.23-1.9] | 0.440 |
| Microbial risk classification | 138 | 1.73 [0.87-3.44] | 0.114 | - | - | - |

Other improved includes boreholes, rainwater, protected dug wells and protected springs.
