# Supplementary material for: Fecal Contamination of Drinking-Water in Low- and Middle-Income Countries: A Systematic Review and Meta-Analysis
Source: PLoS Med. 2014 May 6;11(5):e1001644. doi: 10.1371/journal.pmed.1001644 (PMC4011876; doi:10.1371/journal.pmed.1001644)
Supplement: Table S3 — Variation in microbial safety during the year, findings of included studies for selected source types. (DOCX) [file pmed.1001644.s012.docx]

**Table S3: Variation in microbial safety during the year, findings of selected studies**

| **Source** | **Findings** |
| --- | --- |
| Rain-water | One study in costal Bangladesh found more frequent contamination during the rainy season than the dry season [166]. Another study in India found that all rainwater systems were free of contamination in winter and many were contaminated in summer [335]. |
| Piped | Three studies of municipal water supplies in India show no clear trend for between seasons: in Gangtok, 13 taps were contaminated with TTC during the monsoon whereas none were positive in the summer or winter [182]; in Shillong, non-compliance at consumer endpoints was highest in the summer (100%, n=26) and somewhat lower post-monsoon and in winter (88% and respectively 85%) [184]; in Guwahati, noncompliance was lower post-monsoon (17/49) than summer or winter (31/49, 36/49) [183]. In urban and rural Brazil, Nogueira et al. [243] found treated waters to range from 0% to 6% (taps and reservoirs), with the lowest levels in the summer. Yassin et al. [347] report data from Palestine that show compliance rates ranging from 0-17% for piped water throughout the year, but it is not clear whether these data represent surveillance in the same areas. |
| Boreholes | Hoque et al. [159] found that the proportion of contaminated boreholes varied considerably lower in the dry season (37%) than during or after the monsoon (61%, and 57% respectively). However, the geometric mean and median values of TTC counts at the tubewell sites did not vary significantly between the surveys. Mertens et al [224] note that boreholes with handpumps provided the best quality water throughout the year and were therefore not as susceptible to seasonal trends as other groundwater sources. Potgieiter et al. [277] found significant differences between the dry and rainy seasons TTC compliance for private but not communal boreholes in Limpopo province, South Africa. Ranges were similar, but the levels were somewhat higher (respectively 7.8 to 19.0 and 4.4 to 7.5 per 100 ml). |
| Dug wells | Ejechi et al. [118] found wet season counts to be significantly higher than dry season counts for TTC for both covered and uncovered hand dug wells combined (GM of 3 vs. 14 per 100 ml). Knappett et al. [191] noted substantial variation in the proportion of samples positive for *E. coli* based on weekly grab samples from 35 private wells (30-80% noncompliance). However, the level of contamination was generally low, with the 75^th^ percentile ranging from 1-5 CFU/100 ml. In an area of Sri Lanka where nearly all protected dug wells were contaminated, Mertens et al. [224] found little variation in the proportion of samples with TTC (from 93% to >99%). The geometric mean contamination for the contaminated wells ranged from 50 to 150 per 100 ml and was highest during the monsoon in December. Yassin et al. [347] found that noncompliance levels for wells ranged from 0 to 10%, peaking in August. Razzolini et al. [291] collected water samples from 177 wells every other month. The authors concluded that there was no obvious relationship between rainfall and contamination levels. Geometric mean contamination level for the wells ranged from to around 2 to 50. Karnchanawong et al. [179] found rainy season and dry season ranges to differ (53-55000 vs. 13-5700) for shallow wells near a waste disposal site. |

Note: Several studies did not disaggregate temporal trends by supply type or provided information about the susceptibility of more specific types of wells. For example, Nogueira et al. [243] found greater variation for springs and private wells than for treated piped supplies. The proportion of samples positive for TTC increased with both rainfall and water temperature. A study of various pump designs in Malawi found that the level of contamination was approximately twice as high in the wet season as the dry season [325].
