# Supplementary material for: Fecal Contamination of Drinking-Water in Low- and Middle-Income Countries: A Systematic Review and Meta-Analysis
Source: PLoS Med. 2014 May 6;11(5):e1001644. doi: 10.1371/journal.pmed.1001644 (PMC4011876; doi:10.1371/journal.pmed.1001644)
Supplement: Alternative Language Abstract S1 — Mandarin Chinese translation of the abstract by Hong Yang. (DOCX) [file pmed.1001644.s013.docx]

背景：获得安全饮用水是健康和人权的基本需求。世界卫生组织和联合国儿童基金会采用“使用改善型水源”而没用考虑水质测定作为指标来评估全球安全饮用水的获得情况。我们的研究目标是测定是否“改善型的”水源的饮用水比“非改善型的”水源的饮用水较少含粪便污染，并测定不同的水源和不同环境下污染的种类和程度。

方法和发现：我们搜索和鉴定了在线数据库包括PubMed、Web of Science和灰色文献中的中文、英文、法文、葡萄牙文和西班牙文的文献。本研究只包括文献是发表于1990年到2013年8月，关于中-低收入国家的饮用水，评估了饮用水的大肠埃希氏菌（*E. coli*）或者耐热大肠菌（thermotolerant coliforms， TTC），并且提供了特定的水源。总共有319篇研究，报道了96,737个水样。“改善型”的饮用水与“非改善型”水源的饮用水相比，有较低的污染几率（OR=0.15 [0.10-0.21], I2=80.3% [72.9-85.6]）。但在38%的191个研究中，超过1/4的改善型水源水样含有粪便污染。低收入国家(OR=2.37, [1.52-3.71]; p<0.001)和农村环境(OR=2.37 [1.47-3.81] p<0.001)的水源更可能被污染。很少有研究报道储存水的水质，卫生风险，和有效的随机选择样品。由于采样的次数较少和在饮用前水质恶化，水质安全性可能被高估了。

结论：获得“改善型水源”提供了一个卫生保护的措施，但是并不能确保不受粪便污染，也不能确保在不同水源或者环境下的一致性。因此国际性的评估极大的高估了安全饮用水的使用，而且不能完全反映水获得的不均衡性。新的提高的监测策略将包括卫生保护和水质测定。
