# Supplementary material for: Fecal Contamination of Drinking-Water in Low- and Middle-Income Countries: A Systematic Review and Meta-Analysis
Source: PLoS Med. 2014 May 6;11(5):e1001644. doi: 10.1371/journal.pmed.1001644 (PMC4011876; doi:10.1371/journal.pmed.1001644)
Supplement: Protocol S1 — Systematic review protocol. (DOCX) [file pmed.1001644.s015.docx]

Fecal contamination of drinking water supplies in low and middle income countries: a systematic review and meta-analysis

**Protocol**

Version 2.8, Aug 2013

Authors: Rob Bain^1^, Ryan Cronk^1^, Jim Wright^2^, Hong Yang^2^, Tom Slaymaker^3^ and Jamie Bartram^1^

^1^The Water Institute, University of North Carolina at Chapel Hill

^2^Geography and the Environment, University of Southampton

^3^WaterAid, London, United Kingdom

# Background

The improved drinking-water source indicator has been used to monitor progress toward the Millennium Development Goal (MDG) target 7c, to “halve the proportion of the population without sustainable access to safe drinking-water”. The Joint Monitoring Programme of the World Health Organization (WHO) and UNICEF is responsible for monitoring access to the MDG target. Recognizing that not all improved sources provide the same level of service and the JMP has begun to report progress for different water sources as part of a “water ladder”^[[1]](#footnote-1)^.

Several studies have shown that many improved sources are contaminated with fecal indicators and should be considered unsafe [[1-3](#_ENREF_1)]. There have also been calls for a more graduated approach to assessing the suitability of water sources which balances access, quality, reliability, cost and management [[4](#_ENREF_4)].

In doing so, the safety of both improved and unimproved sources may need to be reassessed.

In January 2012, the Joint Monitoring Programme announced the establishment of four expert working groups on drinking water, sanitation, hygiene and equity and non-discrimination. The outputs of these expert working groups will inform the various on-going political processes led by the UN Secretary General and are expected to culminate in a post-MDG summit at the UN General Assembly in September 2013. The Water Working Group (WWG) is proposing to continue using technology classifications as part of the new definitions of basic and intermediate drinking water services.

This review was commissioned by the Joint Monitoring Programme WWG to investigate the safety afforded by improved sources, how this compares to unimproved sources and to what extent this varies between settings and supply types.

# Definitions

**Non-compliance** The proportion of samples (or sources) in which *E. coli* or thermotolerant coliform (TTC) are detected.

**Risk level** The proportion of samples that are within the concentration ranges <1 (“not detected”) 1-10 (“low”), 10-100 (“moderate”) and >100 (“high”) *E. coli* of TTC per 100 ml.

**Risk score** A summary measure based on a sanitary inspection. The risk score is the percentage of sanitary risks reported for a given water supply as a proportion of the total number of sanitary risks evaluated.

# Objectives

The objectives of this study are to review evidence for the use of facility type classifications as a means to judge safety in global reporting, specifically addressing the following questions:

- Is water from improved sources more frequently compliant with health guidelines for microbial water quality than water from unimproved sources?
- Are microbial compliance levels consistent between (a) different types of water supply, (b) countries or regions and (c) rural and urban areas?
- Are some types of water source associated with higher (a) levels of microbial contamination and (b) risk scores as assessed by sanitary inspection?

The review is restricted to measures of fecal contamination of drinking-water quality, specifically *Escherichia coli* (*E. coli*) or thermotolerant coliform (TTC). We assess compliance according to the WHO Guidelines for Drinking-Water Quality, which recommend the use of *E. coli* or alternatively TTC as a measure of fecal contamination of drinking-water [[5](#_ENREF_5)]. The WHO guideline value is “none detected in any 100-ml sample” [[5](#_ENREF_5)].

The review covers improved sources of drinking-water and “other unimproved” sources of drinking-water such as unprotected wells and tanker trucks. It will also cover packaged waters.

# Methods

This review will be conducted in accordance with the PRISMA statement [[6](#_ENREF_6)].

## Search strategy

Studies will be identified from three sources: peer-reviewed literature, grey literature, and submissions from experts. To identify peer-reviewed literature, the topic “water quality” will be combined with terms to restrict the search to drinking-water and either a measure of microbial water quality or sanitary risk (Box 1; Table 1). We further restrict the search to low and middle-income countries (LMICs) using a list of country names (Table 1). The following databases will be consulted: PubMed, Web of Science, BIOSIS Previews, African Index Medicus, Global Health Library (WHO), SciELO, and Environmental Science and Pollution (including Water Resources Abstracts, Geobase, Microbiology Abstracts A & B, Pollution Abstracts), and three Chinese databases (CNKI, Wangfangdata and Cqvip). See Table 2 for further details.

**Box 1: Search terms**

Search terms were developed and combined using Boolean operators as follows:

(<water quality>) AND (<drinking> OR <supply type>) AND ((<measure of safety>) OR (<measure of sanitary risk>)) AND (<low and middle income country list>)

((water) AND (safe OR quality)) AND ((Drink* OR potable OR bottle* OR supply OR supplies OR source) OR (Household connection OR tap OR faucet OR piped OR pipe OR utility OR reticulated OR endpoint OR standpipe OR spigot OR distribution network OR standpipe OR household connection OR protected well OR unprotected well OR hand pump OR handpump OR rope pump OR dug well OR borehole OR bore OR tubewell OR tube well OR open well OR shallow well OR traditional well OR drilled well OR covered well OR lined well OR rehabilitated well OR windlass OR bore well OR drilled well OR ring well OR hand drawn OR pumped OR communal OR artesian well OR rainwater OR rainjars OR rain harvesting OR rain jar OR Karst OR spring OR Tanker OR vehicle OR Bottle OR bottled OR mineral OR sachet OR cooler OR fountain OR kiosk OR neighbor OR neighbourhood OR bottled OR sachet OR packaged OR hawked OR cistern OR mineral OR water fountain OR water dispenser OR “municipal water” OR tank OR tanker OR truck OR sand-filtered OR pond sand OR cooler OR ponds OR treated system OR vendor OR neighbor OR neighbour OR water station OR kiosk OR jerry can OR storage container OR cistern OR groundwater OR ground)) AND ((coli or coliform OR bacteriological OR microbial OR microbiological OR coliforms OR fecal OR faecal) OR (risk of contamination OR (sanitary AND (risk OR inspection OR survey))))

All results will be limited to publication years between 1990 and 2013, covering the period between the Millennium Development Goals baseline and this review. These terms will be combined with a list of LMICs – see Table 1.

Grey literature will be sourced from a variety of websites including those used in previous WASH-related literature reviews [[7](#_ENREF_7),[8](#_ENREF_8)]. These will include: IRC WASH Library, ProQuest dissertations and theses, UNICEF (evaluations database), UNEP, UN Water Library (which appears to capture most, if not all, UN Organization water documents), USAID, WASH Funders Knowledge Center, Water, Engineering, and Development Centre (WEDC), WaterAid and the World Bank WSP. An email requesting submissions of relevant studies will be distribute to selected water professional networks.

Search terms will be adapted to the databases where required. Simplified terms will be used for the grey literature sites and translations of these terms will be used in regional databases in order to identify studies in Chinese, Portuguese, Spanish and French (Table 3).

Bibliographies of included studies and relevant reviews will be searched. Depending on the resources available, the titles and abstracts from selected journals will be scanned. These journals will be selected based on the number of eligible articles identified from each journal in previous searches.

## Eligibility

Studies will be included in the review provided they: (i) sampled water that is primarily and regularly used for drinking at either the point of collection or consumption^[[2]](#footnote-2)^; (ii) contained extractable data on fecal indicator bacteria, thermotolerant coliform or Escherichia coli; (iii) were published between 1990 and Feb 2013; (iv) included at least 10 separate water samples from different water supplies of a given type or, in the case of reticulated systems, different taps or in the case of packaged waters, number of different brands; (v) reported data from LMICs as defined by the Millennium Development Goals regions; and, (vi) were published in English, French, Portuguese or Spanish; and, (vii) included sufficient detail about the water sources for them to be categorized; and, (viii) would not be classified as surface waters by the JMP. We choose to exclude studies based on total coliform because they can be present or grow in the environment and, as a consequence, the indicator lacks international comparability. Other indicators such as coliphage and direct pathogen detection, although promising fecal indicators have yet to be widely used.

We will not include surface waters as these are known to be highly contaminated and unfit for drinking, often exceeding bathing water quality standards. We include drinking water sources such as bottled water and sachet water that do not form part of the JMP improved source classification because their acceptability is dependent on another source, namely the household’s primary source of water for washing and cooking.

## Study selection

The approach will differ for peer-reviewed and grey literature. Bibliographic software (Endnote) will be used to export all references from online databases of peer-reviewed research to a spreadsheet once duplicates are identified and removed. Study selection will be conducted in two stages: screening of titles and abstracts followed by screening of full texts. For studies in English, independent primary reviews (RB RC) will be recorded in separate spreadsheets and these will be combined prior to the secondary review. Any study selected by either reviewer will proceed to the second stage and, where possible, full texts will then obtained for these studies. The secondary review will be carried out by either reviewer (RB or RC) and reasons for excluding a study will be recorded in a shared Excel spreadsheet.

Grey literature will be screened by one reviewer (RC or RB). Search terms used in each database will be recorded and the number of studies returned will be documented together with the number selected based on the title and/or abstract. Selected studies were included in the spreadsheet with the peer-reviewed literature, with an additional column to indicate their source.

Unfortunately, studies published in Portuguese, Spanish, French (reviewed and screened by RB) and Chinese (reviewed and screened by HY) will not be subject to secondary review or quality control by a second reviewer.

## Data extraction

Basic descriptive data from eligible studies (author, year of publication etc.) will be extracted and we will also extract additional study characteristics thought to influence water quality, including the setting (urban/rural) and season (wet/dry) or dates of sampling. We will classify studies based on study design as this is thought to affect the extent to which they are affected by bias (Table 4); our classification includes: case-control, intervention, cross-sectional survey, longitudinal survey and diagnostic study^[[3]](#footnote-3)^.

Where possible, we will extract the following information for each source type in the studies: non-compliance (presence of *E. coli* or TTC); mean, geometric mean and/or median level of contamination (*E. coli* or TTC per 100 ml); standard deviation, variance or standard errors (*E. coli* or TTC per 100 ml); Risk categories of microbial contamination (<1, 1-10, 10-100 and >100 *E. coli* or TTC per 100 ml); number of samples tested; number of supplies tested^[[4]](#footnote-4)^; risk of contamination score; analytical method used to detect fecal indicator bacteria.

Where levels of contamination were monitored over an extended period or multiple periods, the proportion of samples containing fecal indicator bacteria will be recorded. For studies reporting both *E. coli* and TTC, we use only the *E. coli* results. To explore the influence of seasons, we will record those studies that refer to water quality during “wet”, “rainy” or “dry” periods or equivalent. For intervention studies we will extract either the control group or baseline survey, depending on which is greater number of samples.

For studies including measures of sanitary risk, we will extract information to enable us to calculate the risk score; this will include the number of risks assessed and the proportion found to be present in each group of water supplies. We will also extract any findings comparing sanitary risk to the level of fecal indicator bacteria. We will also identify studies taking water samples from non-domestic settings (schools, markets, health facilities), taking place during or after emergencies, and reporting information on chlorine residual.

Once data had been extracted from all eligible studies, a subset of the English language studies (10%) will be randomly selected for quality control. These will be assigned to RC or RB depending on which of these researchers had originally extracted data on the study. Results will be compared with the originally extracted data and any disagreements will be resolved by agreement between the reviewers (RB RC) or a third reviewer (JW JB).

## Assessment of study quality

Studies will be rated for quality based on criteria outlined in Table 5. A quality rating will be based on the number of affirmative responses, resulting in a point score of between 0 and 12. No study will be excluded based on a low quality score but we will explore the influence of study quality on findings from the review.

## Matching

Results for each type of water supply in a given study will be matched to the JMP water ladder and, where possible, classified as either improved or unimproved.

Where the appropriate match cannot be determined our approach will differ depending on the type of supply. For example, we will:

- Group water sources of a similar type from different geographical regions or those that would fall under the same category in the JMP classification even if these are separately reported in the original study
- Group wells from studies that did not distinguish between protected and unprotected (“not classified dug wells”)
- Grouped studies for other supplies including: packed (or sachet) water, vendor water.
- Differentiate between samples at the point of use (e.g. household storage containers) and point of collection.

We will exclude water supply types if insufficient information is provided, for example if there is no distinction between drilled and dug wells. The matching process will be recorded in the data extraction sheet (including the description of the water source in the original report) and checked by two reviewers (RB RC).

## Analysis

Given that many of the studies of water quality are non-random, there is a high risk of selection bias [[9](#_ENREF_9)]. However, there is growing consensus that non-randomised studies should be included in systematic reviews [[10](#_ENREF_10)]. The review will therefore strike a balance between narrative synthesis and exploratory quantitative analysis. For the quantitative analysis, we will use Stata to implement meta-analysis and meta-regression. Given the anticipated heterogeneity we choose random effects models *a priori* [[11](#_ENREF_11)].

***Heterogeneity.***

We use traditional approaches to investigate heterogeneity between all studies and the following subgroups: water source categories, MDG regions, study designs and country income-level. We will use Higgins I^2^ [[12](#_ENREF_12)] to quantify heterogeneity. We will also aim to identify clusters of studies with similar results or any outliers by visual investigation of study results.

***Assessment of bias.***

We will investigate the extent of bias between studies using standard approaches. Specifically, we will assess the potential for different forms of bias by the following methods:

- The influence of study design will be assessed by sub-group analysis for improved and unimproved sources. Study design is thought to influence the susceptibility to bias in estimates of the non-compliance (see Table 4). In particular, we will examine the difference between cross-sectional and longitudinal measures of microbial contamination as well as comparing random, representative and non-random selection using the study bias indicator.
- Publication bias will be assessed using a funnel plot and Egger’s test. It is thought that there may be a tendency for smaller studies to only be reported if they find high levels of contamination. We will also assess sensitivity of results to study size terciles.
- Study quality rating terciles (“low” “medium” “high”) will be used to assess the whether study quality is related to non-compliance and levels of contamination.
- If data permit, we will compare estimates of water quality in “dry” and “wet” seasons to draw conclusions about the potential for temporally unbalanced studies to bias studies and the direction of their bias.

***Improved vs. unimproved***

Two approaches will be used to compare non-compliance of improved sources to unimproved sources: (i) examining differences across all eligible studies graphically and (ii) for individual studies that include both improved sources and unimproved sources, meta-analysis of the odds ratio for non-compliance.

***Relative safety of source types***

Differences in the levels of microbial contamination and sanitary risk will also be investigated by graphical display of the risk classification for each study in addition to the pooled analyses. We will assess differences in median, mean and geometric mean contamination rates and comment on the comparability of these measures.

***Differences between regions and countries***

To explore differences between countries, we will contrast contamination between MDG regions and assess the extent to which differences are observed for higher quality randomized studies. We will also compare countries by income-level. We will investigate differences between urban and rural areas.

***Additional sub-group analyses***

If possible, in addition to the above sub group analyses, we will investigate: (i) the difference between water at the point of use and at the point of collection, (ii) water quality in non-domestic setting or emergency settings.

# Plans for updating the review

Related reviews will be based on a subset of the data. Revised or expanded versions of the review will be subject to the availability of funding. The database generated from this study will be shared with the WHO and UNICEF.

# Acknowledgements

This research is funded by the Water Working Group of the Joint Monitoring Programme. We thank Mellanye Lackey for support in defining the search strategy.

# Conflict of interest

None declared

# Tables

**Table 1: Search terms in English**

| (water) AND (safe OR quality)  AND  ((Drink* OR potable OR bottle* OR supply OR supplies OR source) OR (Household connection OR tap OR faucet OR piped OR pipe OR utility OR reticulated OR endpoint OR standpipe OR spigot OR distribution network OR standpipe OR household connection OR protected well OR unprotected well OR hand pump OR handpump OR rope pump OR dug well OR borehole OR bore OR tubewell OR tube well OR open well OR shallow well OR traditional well OR drilled well OR covered well OR lined well OR rehabilitated well OR windlass OR bore well OR drilled well OR ring well OR hand drawn OR pumped OR communal OR artesian well OR rainwater OR rainjars OR rain harvesting OR rain jar OR Karst OR spring OR Tanker OR vehicle OR Bottle OR bottled OR mineral OR sachet OR cooler OR fountain OR kiosk OR neighbor OR neighbourhood OR bottled OR sachet OR cistern OR mineral OR water fountain OR water dispenser OR “municipal water” OR tank OR tanker OR truck OR sand-filtered OR pond sand OR cooler OR ponds OR treated system OR vendor OR neighbor OR neighbour OR water station OR kiosk OR jerry can OR storage container OR cistern OR groundwater OR ground))  AND  ((coli OR bacteriological OR microbial OR microbiological OR fecal OR faecal) OR (risk of contamination OR (sanitary and (risk or inspection or survey))))  AND  (Afghanistan or Algeria or Angola or Anguilla or Antigua or Barbuda or Argentina or Armenia or Armenian or Aruba or Azerbaijan or Bahamas or Bahrain or Bangladesh or Barbados or Benin or Byelarus or Byelorussian or Belarus or Belorussian or Belorussia or Belize or Bhutan or Bolivia or Botswana or Brazil or Brunei or Burkina Faso or Burkina Fasso or Upper Volta or Burundi or Urundi or Cambodia or Khmer Republic or Kampuchea or Cameroon or Cameroons or Cameron or Camerons or Cape Verde or Cayman Islands or Central African Republic or Chad or Chile or China or Colombia or Comoros or Comoro Islands or Comores or Mayotte or Congo or Zaire or Cook Islands or Costa Rica or Cote d'Ivoire or Ivory Coast or Croatia or Cuba or Cyprus or Djibouti or French Somaliland or Dominica or Dominican Republic or East Timor or East Timur or Timor Leste or Ecuador or Egypt or United Arab Republic or El Salvador or Eritrea or Ethiopia or Falkland Islands or Las Malvinas or Fiji or Gabon or Gabonese Republic or Gambia or Gaza or Georgia Republic or Georgian Republic or Ghana or Gold Coast or Greece or Grenada or Guatemala or Guinea or Guam or Guadeloupe or Guiana or Guyana or Haiti or Honduras or Hong Kong or India or Maldives or Indonesia or Iran or Iraq or Jamaica or Jordan or Kazakhstan or Kazakh or Kenya or Kiribati or Korea or Kosovo or Kuwait or Kyrgyzstan or Kirghizia or Kyrgyz Republic or Kirghiz or Kirgizstan or Lao PDR or Laos or Lebanon or Lesotho or Basutoland or Liberia or Libya or Macau or Madagascar or Malagasy Republic or Maldives or Malaysia or Malaya or Malay or Sabah or Sarawak or Malawi or Nyasaland or Mali or Malta or Marshall Islands or Martinique or Mauritania or Mauritius or Agalega Islands or Mexico or Micronesia or Middle East or Mongolia or Montserrat or Morocco or Ifni or Mozambique or Myanmar or Myanma or Burma or Namibia or Nauru or Nepal or Niui or Netherlands Antilles or New Caledonia or Nicaragua or Niger or Nigeria or Northern Mariana Islands or Oman or Mayotte or Muscat or Pakistan or Palau or Palestine or Panama or Paraguay or Peru or Philippines or Philipines or Phillipines or Phillippines or Polynesia or Puerto Rico or Qatar or Reunion or Rwanda or Ruanda or Saint Kitts or St Kitts or Nevis or Saint Lucia or St Lucia or Saint Vincent or St Vincent or Grenadines or Samoa or Samoan Islands or Navigator Island or Navigator Islands or Sao Tome or Saudi Arabia or Senegal or Serbia or Montenegro or Seychelles or Sierra Leone or Singapore or Sri Lanka or Ceylon or Solomon Islands or Somalia or South Africa or Sudan or Suriname or Surinam or Swaziland or Syria or Tajikistan or Tadzhikistan or Tadjikistan or Tadzhik or Tanzania or Thailand or Togo or Togolese Republic or Tokelau or Tonga or Trinidad or Tobago or Tunisia or Turkey or Turkmenistan or Turkmen or Turks Caicos or Tuvalu Uganda or United Arab Emirates or Uruguay or Uzbekistan or Uzbek or Vanuatu or New Hebrides or Venezuela or Vietnam or Viet Nam or Virgin Islands OR West Bank or Yemen or Yugoslavia or Zambia or Zimbabwe) |
| --- |

**Table 2: Databases selected for a review of water quality studies**

| **Type** | **Name** |
| --- | --- |
| **Database** | PubMed, ISI Web of Science, BIOSIS Previews, Global Health Library (African Index Medicus, LILACS), SciELO,Public Health, Environmental Science and Pollution (including Water Resources Abstracts, Geobase, Microbiology Abstracts A & B, Pollution Abstracts), Environment complete, Cairn (cairn.info).  CNKI, Wangfangdata and Cqvip (in Chinese only). |
| **Grey literature** | IRC WASH Library, ProQuest dissertations and theses, UNICEF (including evaluations database), UNEP, UN Water Library, USAID, WASH Funders Knowledge Center, Water, Engineering, and Development Centre (WEDC), WaterAid, World Bank Water and Sanitation Programme, the US Environmental Protection Agency DRINK facility, Pan American Health Organization (PAHO) library catalog, OpenSigle, the online catalogue of the International Development Research Centre (IDRC), Canada. |

**Table 3: Simplified search terms and translations used**

| **English** | **Spanish** | **Portuguese** | **Chinese** | **French** |
| --- | --- | --- | --- | --- |
| Water AND Quality | Calidad AND agua | Qualidade AND água | 水质 | Qualité AND Eau |
| Potable OR Drinking OR Supply OR supplies | Potable OR beber OR bebida OR Fuente | Potável OR beber OR bebida OR Fonte | 饮用水 OR  供应 OR  供应系统 | Potable OR boire OR boisson OR Source |
| Coli OR (Sanitary AND (risk OR inspection OR survey)) | Coli OR (Sanitari* AND (riesgo OR encuesta OR inspección)) | Coli OR (Sanitári* AND (Risco OR inspeção)) | 大肠菌 OR（卫生 AND  (风险 OR  调查) - | Coli OR (sanitaire AND (inspection OR evaluation OR contrôle OR Risqué)) |

**Table 4: Study designs and anticipated sources of bias for compliance and contamination levels**

| **Study design** | **Anticipated bias for microbial compliance** | **Anticipated b­ias for microbial contamination level** |
| --- | --- | --- |
| Intervention | Underestimate, disease or water quality focused | Overestimate, disease or water quality focused |
| Case-control | Underestimate, sites chosen for disease status | Overestimate, sites chosen for disease status |
| Cross-sectional survey (not randomized) | Unclear, one-off sampling likely to overestimate compliance. Accessibility considerations may result in the selection low contamination sites whereas sites may be chosen which have poor water quality. | Unclear, accessibility considerations may result in the selection low contamination sites whereas sites may be chosen which have poor water quality. Will depend on timing of sampling. |
| Longitudinal survey (not randomized) | Unclear, will depends on intensity of sampling and how sites are chosen. | Unclear, will depend on timing of sampling and how sites are chosen |
| Cross-sectional survey (not randomized) | Overestimate, one-off sampling likely to overestimate compliance | Unclear, may depend on season and |
| Longitudinal survey (not randomized) | Least susceptible to bias | Dependent on the metric used |
| Diagnostic study | Unclear, sites typically chosen to provide a range of water quality levels. | Unclear, sites typically chosen to provide a range of water quality levels. |

**Table 5: Quality criteria used to assess studies of microbial water quality.**

| **Criterion** | **Question** |
| --- | --- |
| Selection described | Do the authors describe how the water samples were chosen, including how either the types of water source or their users were selected? |
| Selection  Representative | Did the authors detail an approach to designed to provide representative picture water quality in a given area? |
| Selection  randomized | Was sampling randomized over a given study area or population? |
| Region described | Does the study report the geographic region within the country where it was conducted? |
| Season reported | Were the seasons or months of sampling reported? |
| Quality control | Were quality control procedures specified or referred to? |
| Method described | Are well-defined and appropriate methods of microbial analysis described or referenced? |
| Point of sampling | Was the point at which water was sampled well-defined? (For example whether the water was collected from within a household storage container or directly from a water source) |
| Handling described | Are sample handling procedures are described, including sample collection, transport method and duration? |
| Handling minimum criteria | Does sample handling and processing meet the following criteria: transport on ice or between 2-8**°**C, analysis within six hours of collection and specified incubation temperature? |
| Accredited laboratory | Was the microbial analysis conducted in an accredited laboratory setting? |
| Trained technician | Do the authors state whether trained technicians conducted the water quality assessments or the analyses where undertaken by laboratory technicians? |
| External review | Was the study subject to peer review or external review prior to publication? |

# References

1. Bain RES, Gundry SW, Wright JA, Yang H, Pedley S, et al. (2012) Accounting for water quality in monitoring access to safe drinking-water as part of the Millennium Development Goals: lessons from five countries. Bull World Health Organ 90: 228-235A.

2. Onda K, LoBuglio J, Bartram J (2012) Global access to safe water: accounting for water quality and the resulting impact on MDG progress. Int J Environ Res Public Health 9: 880-894.

3. Godfrey S, Labhasetwar P, Wate S, Pimpalkar S (2011) How safe are the global water coverage figures? Case study from Madhya Pradesh, India. Environmental Monitoring and Assessment 176: 561-574.

4. Bartram J (2008) Improving on haves and have-nots. Nature 452: 283-284.

5. WHO (2011) Guidelines for Drinking-Water Quality. Geneva: WHO.

6. Moher D, Liberati A, Tetzlaff J, Altman DG, Group P (2009) Preferred reporting items for systematic reviews and meta-analyses: the PRISMA statement. PLoS Med 6: e1000097.

7. Opryszko MC, Huang H, Soderlund K, Schwab KJ (2009) Data gaps in evidence-based research on small water enterprises in developing countries. J Water Health 7: 609-622.

8. Wright J, Gundry S, Conroy R (2004) Household drinking water in developing countries: a systematic review of microbiological contamination between source and point-of-use. Trop Med Int Health 9: 106-117.

9. Egger M, Schneider M, Davey Smith G (1998) Spurious precision? Meta-analysis of observational studies. BMJ 316: 140-144.

10. Higgins JPT, Ramsay C, Reeves BC, Deeks JJ, Shea B, et al. (2013) Issues relating to study design and risk of bias when including non-randomized studies in systematic reviews on the effects of interventions. Research Synthesis Methods 4: 12-25.

11. Borenstein M (2009) Introduction to meta-analysis. Chichester, U.K.: John Wiley & Sons. xxviii, 421 p. p.

12. Higgins JP, Thompson SG, Deeks JJ, Altman DG (2003) Measuring inconsistency in meta-analyses. BMJ 327: 557-560.

1. Definitions of the water ladder and improved sources are available from the Joint Monitoring Programme website: wssinfo.org [↑](#footnote-ref-1)
2. In more detail: we will not include samples taken from within water treatment plants, at points in piped distribution systems other than the consumer tap or groundwater samples from studies that indicate this is first treated before distribution (e.g. production wells), [↑](#footnote-ref-2)
3. We differentiate between cross-sectional and longitudinal as follows: Longitudinal defined as any study with at least 6 months and more than two repeated samples at each water point. Some studies combine features of more than one study designs. We categorize in the following priority order: case-control, intervention, diagnostic study, cross-sectional survey, longitudinal survey. [↑](#footnote-ref-3)
4. In the case of piped systems, the number of consumer endpoints. In the case of branded waters (bottled, sachet) the number of brands. In the case of household stored water, the number of households. [↑](#footnote-ref-4)
